# 

**Published:** 1894-01

**Authors:** 


### CONTENTS—JANUARY, 1894.—VOL. IX., NO. 7.

ORIGINAL COMMUNICATIONS—

Uterine Hemorrhages; by Prof. Wm. Keiller, F. R. C. S., Ed., Galveston . . . . . . . . . . . . . . . . . . . . . . . . . . . . . 313

Some Observations on Puerperal Eclampsia; by Sam. Cunningham, M. D., Elgin . . . . . . . . . . . . . . . . . . . . . . . . . . 320

Discussion on Dr. Cunningham's paper . . . . . . . . . . . . . . 327

The Code of Ethics; by M. DeCausey, M. D., Sealy . . . . . . . . . 328

Varicocele and Its Radical Cure; by Sam'l E. Milliken, New York . 332

ABSTRACTS OF CURRENT MEDICAL LITERATURE—

Department of Gynecology; edited by Prof. Wm. Keiller, F. R. C. S., Ed., Galveston . . . . . . . . . . . . . . . . . . . . . . . . . 334

Department of Therapeutics; edited by Prof. D. Cerna, M. D., Galveston . . . . . . . . . . . . . . . . . . . . . . . . . . . . . 338

SOCIETY NOTES—

Galveston County Medical Society, Minutes, 343. Discussion on Uterine Hemorrhages, 344. Central Texas Medical Society, Jan'y Meeting, 346. Austin District Med. Society, Annual Meeting, . 343-357

EDITORIAL—

Ex-Confederates Disfranchised, 347. Resolution by Austin District Medical Society, 349. Necrological: Dr. Wagley, Dr. Paine, Dr. DeSteiger, 350; Dr. Waters, 353 . . . . . . . . . . . . . . . . . 347-353

MEDICAL NEWS AND MISCELLANY—

An Unusually Full Collection of News Items of Particular Interest to Physicians, 352-7; In Memoriam: Dr. Wagley, 358; Section of Railway Surgery in Medico Legal Society, 359 . . . . . . . . . 352-359

BOOK NOTICES—

Dictionary of Medical Science 362. Text-Book of Medicine, 363. Electro-Therapeutics, 364. The Bacterial Poisons, 364 . . . . 362-364

PUBLISTHERS' NOTES . . . . . . . . . . . . . . . . . . . . . . . 365

